# Recent Advances in Morphological Cell Image Analysis

**DOI:** 10.1155/2012/101536

**Published:** 2012-01-09

**Authors:** Shengyong Chen, Mingzhu Zhao, Guang Wu, Chunyan Yao, Jianwei Zhang

**Affiliations:** ^1^College of Computer Science and Technology, Zhejiang University of Technology, Hangzhou 310023, China; ^2^College of Information Engineering, Zhejiang University of Technology, Hangzhou 310023, China; ^3^Guangxi Academy of Sciences, 98 Daling Road, Nanning 530007, China; ^4^DreamSciTech Consulting, Shenzhen 518054, China; ^5^Department of Informatics, University of Hamburg, 22527 Hamburg, Germany

## Abstract

This paper summarizes the recent advances in image processing methods for morphological cell analysis. The topic of morphological analysis has received much attention with the increasing demands in both bioinformatics and biomedical applications. Among many factors that affect the diagnosis of a disease, morphological cell analysis and statistics have made great contributions to results and effects for a doctor. Morphological cell analysis finds the cellar shape, cellar regularity, classification, statistics, diagnosis, and so forth. In the last 20 years, about 1000 publications have reported the use of morphological cell analysis in biomedical research. Relevant solutions encompass a rather wide application area, such as cell clumps segmentation, morphological characteristics extraction, 3D reconstruction, abnormal cells identification, and statistical analysis. These reports are summarized in this paper to enable easy referral to suitable methods for practical solutions. Representative contributions and future research trends are also addressed.

## 1. Introduction

Cell morphology has become a standard theory for computerized cell image processing and pattern recognition. The purpose of which is the quantitative characterization of cell morphology, including structure and inner-components analysis for better understanding functioning and pathogenesis associated with malignancy and behavior [1].

Morphological cell analysis is a key issue for abnormality identification and classification, early cancer detection, and dynamic changes analysis under specific environmental stress. The quantitative results and primary, objective, and reliable, which is beneficial to pathologists in making the final diagnosis and providing fast observation and automated analysis systems.

In the present study, advances in morphological cell analysis are briefly reviewed. Overall, significant progress has been made in several issues. Morphological cell analysis has been integrated in new methods for biomedical applications, such as automatic segmentation and analysis of histological tumour sections [2–4], boundary detection of cervical cell nuclei considering overlapping and clustering [5, 6], the granules segmentation and spatial distribution analysis [7], morphological characteristics analysis of specific biomedical cells [8–10], understanding the chemotactic response and drug influences [11–14], or identifying cell morphogenesis in different cell cycle progression [15].

Morphological feature quantification for grading cancerous or precancerous cells is especially widely researched in the literature, such as nuclei segmentation based on marker-controlled watershed transform and snake model for hepatocellular carcinoma feature extraction and classification, which is important for prognosis and treatment planning [16], nuclei feature quantification for cancer cell cycle analysis [17], and using feature extraction including image morphological analysis, wavelet analysis, and texture analysis for automated classification of renal cell [18].

Computerized/automated early cancer or abnormalities detection provides a basis for reducing deaths and morbidity, especially for cervical cancer, which is reported to be the most preventable disease through early detection [19], provision of prompt advice, and opportunities for follow-up treatments. As an example, [20] presents a prototype expert system for automated segmentation and effective cervical cancer detection, providing primary, objective, and reliable diagnostic results to gynaecologists in making the final diagnosis. These advances will contribute to realize computer-assisted, interactive, or automated processing, quantification, statistic analysis, and diagnosis systems for biomedical applications.

The scope of this paper is restricted to morphological cell analysis by image processing in the field of biomedical research. Although this topic has attracted researchers as since early as the 1980s [21–23], this survey concentrates on the contributions of the last 5 years. No review of this nature can possibly cite each and every paper that has been published. Therefore, we include only what we believe to be representative samples of important works and broad trends from recent years. In many cases, references were provided to better summarize and draw distinctions among key ideas and approaches.

The paper has five more sections.  Section 2 briefly provides an overview of related contributions.  Section 3 introduces the typical formulation of cell morphology.  Section 4 lists the relevant tasks, problems, and applications of cell morphology.  Section 5 concentrates typical solutions and methods.  Section 6 is a discussion of our impressions on current and future trends.  Section 7 is the conclusion.

## 2. Overview of Contributions

### 2.1. Summary

**Figure 1:**
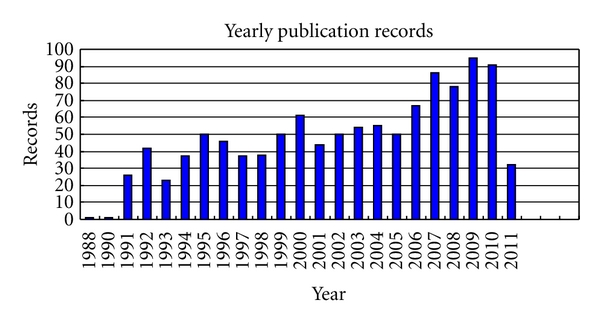
Yearly published records from 1990 to 2010.

From 1980s to 2010, about 1000 research papers with topics on or closely related to morphological cell analysis for robot vision were published.  Figure 1 shows the yearly distribution of these published papers. The plot shows that the topic of morphological cell analysis steadily developed in the past 20 years.

### 2.2. Representatives

Morphological cell analysis has many applications in biomedical engineering. Their most significant roles are summarized as follows.

1. Malignant cell identification and cancer detection [20, 24, 25].
2. Morphological changes during a cell cycle as division, proliferation, transition, and apoptosis [26–28] or to follow cell culture development [29].
3. Morphological differences to elucidate the physiological mechanisms [30] or classify a set of cell populations with different functions such as neurons [31, 32].
4. Dynamic characteristics investigation under specific environmental stress for personalized therapy [33–36] or for the selection of new drugs [37].
5. Morphometrical study such as subcellular structures (DNA, chromosome) analysis [38] for higher animals or plants based on 3D reconstruction [39, 40].

The commonly researched topics for solving morphological problems are listed below.

1. Mathematical morphology theory used in binary, gray, and color images for preprocessing or features analysis [41–48].
2. Location determination: objects located and analysis of distribution [7, 49, 50].
3. Meaningful areas segmentation: based on the features of pixel, edge, region, and model [2–4].
4. Characteristics quantification: based on cytopathology and the experience of physicians [51–58].
5. Recognition, classification automated analysis, and diagnosis [6, 16, 24, 51, 59].

Morphological analysis has become a powerful mathematical tool for analyzing and solving cell informatics. Automatic features quantification is undoubtedly the most widely used estimation technique in this topic. Among the variety of developed methods, the main differences and remarkable features can be summarized briefly: shape, geometrical, intensity, and texture. A few representative types of segmentation and classification are selected for easy appreciation of state-of-the-art as shown in Table 1.

## 3. The Problem and Fundamental Principle

**Figure 2:**
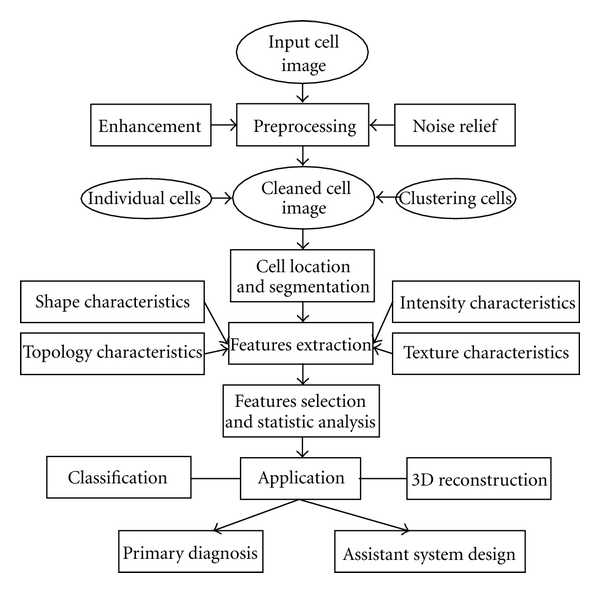
The general procedure of cell image analysis.

The fundamental principle of morphological cell analysis is dependent on cell biology, cytopathology, and the diagnostic experience of pathologists. To study cell characteristics, detect abnormalities, and determine the malignant degree, the pathologists examine biopsy material under a microscope, which is subjective, laborious, and time consuming. Therefore quantitative cell morphology is studied and computer-assisted systems are presented for diagnostic process at the same time. The general procedure of such applications can be described in Figure 2.

## 4. Tasks and Problems

### 4.1. Morphological Operation

Mathematical morphology is the basic theory for many image processing algorithms, which can also extract image shape features by operating with various shape-structuring elements [60]. This processing technique has proved to be a powerful tool for many computer-vision tasks in binary and gray scale images, such as edge detection, noise suppression, image enhancement, skeletonization, and pattern recognition, [45]. This technique is consisted of two parts: binary morphology and gray-scale morphology, and the commonly used operations as morphological dilation and erosion are defined as follows, respectively:

(1) $$\begin{array}{c} \left(f⊕k\right)\left(x,y\right)=\max\left\{f\left(x-m,y-n\right)+k\left(m,n\right)\right\}\text{,}\\ \left(f\Theta k\right)\left(x,y\right)=\max\left\{f\left(x-m,y-n\right)-k\left(m,n\right)\right\}, \end{array}$$

where *f* is the original image (gray scale or binary), which is operated by the corresponding structuring element *k*, and (*x*, *y*) is the pixel of image *f*, (*m*, *n*) is the size of element *k*. After morphological operation, image shape features such as edges, fillets, holes, corners, wedges, and cracks can be extracted.

 Mathematical morphology can also be used in color images avoiding the loss of information of traditional binary techniques [45]. The new operations are based on the order in multivariate data processing.

### 4.2. Cell Localization

Determination of the orientation of a cell, termed localization, is of paramount importance in achieving reliable and robust morphological analysis. Achieving high-level tasks such as segmentation and shape description is possible if the initial position is known. From the early literature, primary methods were used in sample images, such as [61] using a sequence of morphological image operations to identify the cell nuclei and [29] using conditional dilation techniques to estimate unbiasedly cell density and obtain precisely cell contours. The results were acceptable only in single images without any complex factors.

**Figure 3:**
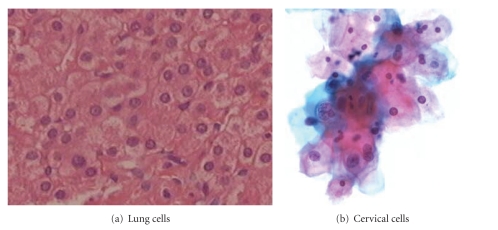
Biomedical cell images.

Even when membranes are partially or completely not visible in the image (Figure 3(a)), the approximate locations of cells can be detected by reconstructing cellular membranes [51]. This method is effective for lung cells location in immunohistochemistry tissue images. Cell nuclei that are in cell clusters detecting are the key point for eliminating the positions of cervical cells in conventional Pap smear images (Figure 3(b)). To deal with this problem, Plissiti et al. present a fully automated method [6]. It takes the advantage of color information to obtain the candidate nuclei centroids in the images and eliminate the undesirable artifacts by applying a distance-dependent rule on the resulted centroids and classification algorithms (fuzzy C-means and support vector machines). The experiments shows that even in the case of images with high degree of cell overlapping, the results are very promising.

 For automatic detection of granules in different cell groups and statistical analysis of their spatial locations, the existing image analysis methods, such as single threshold, edge detection, and morphological operation, cannot be used. Thus, the empirical cumulative distribution function of the distances and the density of granules can be considered [7]. Jiang et al. propose a machine learning method [62], which is based on Haar feature (which is the combination of the intensity, shape, and scale information of the objects), to detect the particle's position.

### 4.3. Segmentation

Segmentation is one of the most important points for automated image analysis and better cell information understanding. The algorithms that have been presented can be divided into edge-based, region-based, and model-based modules. Region-based approaches attempt to segment an image into regions according to regional image data similarity (or dissimilarity), such as scale-space filtering, watershed clustering [63], gray-level threshold [26], and region growing [64]. For clear stained images, multilevel thresholds are the most simply and commonly applied methods for low-level segmentation to remove noise and obtain the interest region (nucleus, cytoplasm, or the whole cell), which are defined as follows:

(2) $$g\left(x,y\right)=\{\begin{array}{cc} I_{i}, & T_{i-1}\leq f\left(m,n\right)\leq T_{i},\\ 0, & \text{others}, \end{array}$$

where *i* is the number of regions need to be divided, *T*_*i*_ is the threshold and the extension ranges from *T*_*i*−1_ to *T*_*i*_ corresponding to the region *i*.

Nevertheless numerous algorithms have been developed, overlapping and connected cluster is still the key problem in cell image segmentation. The methods presented available to solve specific images with clear stained situation, semiautomated algorithms based on preknowledge for adequate segmentation of cell images under complex situation, are always more efficient than totally automated methods.

### 4.4. Quantitative Measurement of Meaningful Parameters

The quantitative measurement of cell features is meaningful for both image segmentation and abnormalities detection. Fast, reproducible, accurate, and objective measurement of cell morphology is beneficial to avoid subjective and interobserver variations, which result in diagnostic shifts and consequently disagreement between different interpreters [20]. The quantitative characteristics of cell or nuclear structure alterations extracted after robust image processing algorithms and 3D reconstruction is also called morphological biosignatures, which learn about cellular level features and nuclear structure including inner-components analysis, such as the quantitative evaluation of the approximate number of mRNA varying during cell cycle, developing, aging, and in different pathologies and treatment with drugs by extracting morphological parameters (cytoplasm and nucleus areas) [28]. Accurate quantification of these parameters could be beneficial for developing robust biosignatures for early cancer detection [1]. Multivariate statistical analyses of morphological data to suggest that quantitative cytology may be a useful adjunct to conventional tests for the selection of new drugs with differentiating potential [37].

The extracting features as cell area, perimeter, centroid, and the length of major and minor axes for calculating more meaningful parameters such as displacement, protrusiveness, and ellipticity, are used to analyze the dynamic changes of human cancerous glioma cells [35], which can also be used to identify different classed of neurons and relate neural structure (such as total dendritic length and dendritic field area) to function [31].

The most meaningful parameters are obtained in discriminating different patterns, such as cell size, shape distribution, and nuclear-to-cytoplasmic ratio for normal and precancerous cervical squamous epithelium determination [44], and texture quantification as a measurement to interchromosome coarseness to study cell proliferation [38]. Local gray level differences and cell density combining with other morphological parameters are possible to follow cell culture development under various experimental conditions [29]. Hitherto, the relationship between malignancy-associated morphological features in single tumour cells and the expression of markers indicating functional properties of these cells remained widely unknown [65].

### 4.5. Statistical Analysis

Multivariate statistic analysis is applied to compare multivariate data and establish the quantitative changes and differences between groups under investigation on their characteristics. The kernel approach is to find a high correlation feature set without redundancy. Principal components analysis (PCA) displays the original variables in a bidimensional space, thus reducing the dimensionality of the data and allowing the visualization of a large number of variables into a two-dimensional plot [11, 49, 66].

## 5. Methods and Solutions

### 5.1. Formulation in Morphological Analysis

Morphological analysis is often studied as the shape appearances of objects and the surfaces of the images, with intensity seen as height and texture appearing as relief. Formulization of morphological features is of benefit to computerized calculation and more efficient than manual morphological quantification, which is still laborious and subjective. The morphology characteristics can be described by shape, geometrical, intensity, and texture analysis.

**Figure 4:**
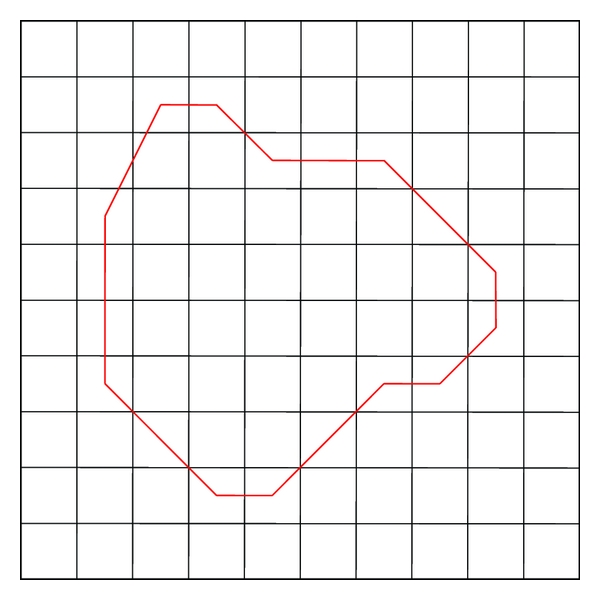
Geometrical features quantification.

The geometrical features of regions can be described by area, radii, perimeter, the major and the minor axis length, and so forth. The area of the object is calculated as the number of pixels of the region (Figure 4, the area defined by the closed curve). Radii are calculated based on projected cell area supposing that each cell is circular. The major and the minor axis length are the maximal and minimum numbers of pixels of the axis, respectively. Take Figure 4 as an example, the perimeter is calculated as follows:

(3) $$P=N_{1}+N_{2}+\sqrt{2}N_{3},$$

where, *N*_1_, *N*_2_, *N*_3_ are the numbers of the horizontal, vertical bevel lines on the boundary, respectively. 

Circularity, rectangle, eccentricity, and irregularity are used to describe the shape features. Circularity (*C*) and rectangle (*R*) represent the rotundity-like and rectangle-like degree, defined as follows:

(4) $$\begin{array}{c} C=\frac{P^{2}}{4\pi A},\\ R=\frac{\text{Area}}{H*W}. \end{array}$$

Eccentricity is defined as follows:

(5) $$\begin{array}{cc} E & =\frac{\text{The}\;\text{minor}\;\text{axis}\;\text{length}}{\text{The}\;\text{major}\;\text{axis}\;\text{length}}. \end{array}$$

Texture is an important visual cue and widely exists in images. Texture feature extraction is the most basic problem for texture analysis including classification and segmentation. Dimension, discrimination, stability, and calculation are considered in practical application and studied for more than fifty years. Based on the statistical theory, structure, model, and signal processing, many effective methods were presented for different applications. Among which, gray level co-occurrence matrix (GLCM) has become one of the best known and most widely used statistic method for texture feature extraction [26], especially in cell image texture feature analyzing. The interrelationship of textural primitives which define morphological texture can be estimated by quite different descriptors, the discriminant value of which varies considerably [67]. The descriptors based on GLCM are summarized in Table 2.

The intensity feature is characterized by the average of the intensity value of all the pixels of the region. For RGB color images, it is calculated independently from the red, green, and blue component of the original image. Histogram is an efficient way to show intensity features. Kruk et al. characterize the histograms of different color components by applying the following parameters: the mean, standard deviation, skewness, kurtosis, maximum value, and the span of the histogram [59].

### 5.2. Deformable Models

It is known that biomedical images are always under complex situation, which made segmentation a hard task for the extraction of the interest region. Because of the various challenges in medical image processing, deformable models were widely investigated and innovated, becoming a powerful tool for medical image segmentation. Active counter model is one of the most classical algorithms. Techniques based on active contour models have the potential to produce better estimates of cell morphologies.

The existing active contour models can be categorized into two classes: edge-based models [68], and region-based models [69]. On one hand edge-based model directly uses intensity gradient information to attract the contour toward the object boundaries. Therefore this kind of model has worse performance for weak object boundaries since cell image exhibits great fuzzy degree due to low contrast at the location of the cell membrane. On the other hand region-based model aims to identify each region of interest by using a certain region descriptor. It guides the motion of the contour, and is less sensitive to the location of initial contours in some extents. It is much more suitable for cell segmentation than the fore one.

Chan and Vese model [70] is one of the most popular region-based active contour models. This model has been successfully used for segmenting images. Chan and Vese model proposed an active contour model that segments an image into two sets of possibly disjoint regions, by minimizing a simplified Mumford-Shah functional. The basic idea is as follows. Assume that *Ω* ⊂ *R* ^2^ is the image domain and *I*: *Ω* → R is a given image. Mumford and Shah consider image segmentation as a problem of seeking an optimal contour *C* that divides the image domain into two approximately piecewise-constant regions with intensities *u*_*i*_ and *u*_0_. Let *C* denote its boundary. Thus the global data fitting term in the Chan and Vese model is defined as follows:

(6) $$E^{cv}\left(c_{1},c_{2}\;\right)=\overset{}{\underset{\bar{\Omega }}{\int }}{\left(I-c_{1}\right)}^{2}dxdy+\overset{}{\underset{\Omega }{\int }}{\left(I-c_{2}\right)}^{2}dxdy,$$

where *Ω* and $\bar{\Omega }$ represent the regions outside and inside the contour *C*, respectively, *c*_1_ and *c*_2_ are two constants that fit the image intensities outside *C* and inside *C*.

This model considers pixels within the same region having the most similarity, and makes up the shortcomings of edged etector. When the contour accurately captures the object boundary, the two fitting terms minimize the fitting energy value. In each segmented area, the clustered pixels' mean value approximately equals the *c*_1_ and *c*_2_, respectively. Thus the fitting terms with respect to *c*_1_ and *c*_2_ are the driving forces that evolve the curve motion on the principle of inner-region homogeneity.

Since the regional difference is the guideline in image segmentation, the interregional differences should be considered as the model's driving force as follows:

(7) $$E=-\frac{1}{2}{\left(c_{1}-c_{2}\right)}^{2}.$$

This kind of region-based active contour model's energy is characterized by the maximum dissimilarity between regions. Minimizing the energy *E* in (7) is the same as maximizing the difference between different regions. Equation (7) formulates the global instructive guidance term.

### 5.3. Classification

The extracted features involved the input to classification procedure for better analysis, correct grading, and pattern recognition. From the literature, unsupervised (as *K*-means and spectral clustering) and supervised (as super vector machine, SVM) classification schemes and artificial neural network (ANN) architecture were applied. SVM clustering is a state-of-the-art method, which was originally proposed in [71]. The decision function of a two-class problem can be written as follows:

(8) $$f\left(x\right)=\omega \cdot \phi \left(x\right)+b=\overset{N}{\underset{i=1}{\sum }}\alpha_{i}y_{i}K\left(x,x_{i}\right)+b,$$

where *x*_*i*_ ∈ *R* ^*d*^ is the sample and *y*_*i*_ ∈ {±1} is the class label of *x*_*i*_. A transformation *ϕ*(·) maps the data points *x* of the input space *R* ^*d*^ into a higher-dimensional feature space *R* ^*D*^, (*D* ≥ *d*). *K*(·, ·) is a kernel function, which defines an inner product in *R* ^*D*^. *K*(·, ·) is commonly defined as follows:

(9) $$\begin{array}{c} K\left(x,x_{i}\right)={\left[\left(x\cdot x_{i}\right)+1\right]}^{q},\\ K\left(x,x_{i}\right)=\exp\left\{-\frac{{\left|x-x_{i}\right|}^{2}}{\sigma^{2}}\right\},\\ K\left(x,x_{i}\right)=\tanh\left(v\left(x\cdot x_{i}\right)+c\right). \end{array}$$

**Figure 5:**
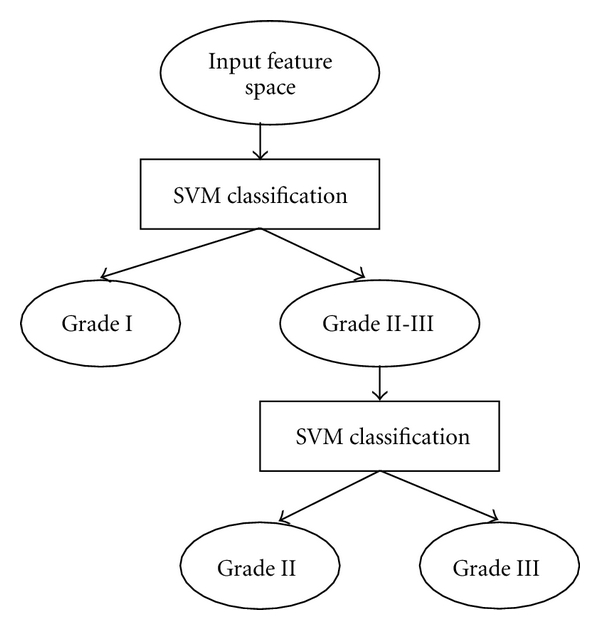
A decision-tree SVM classification scheme.

The parameters *α*_*i*_ ≥ 0 are optimized by finding the hyperplane in feature space with maximum distance to the closest image *ϕ*(*x*_*i*_) from the training set. For multilevel classification based on SVM, a decision-tree classification scheme discriminated between different grades is showed in Figure 5.

Although SVM is one of the most famous methods for classification and has achieved a great success in pattern recognition, problems still exist, such as the neglect of different data distributions within classes. Recently, structural super vector machine (SSVM) is proposed accordingly, which firstly exploits the intrinsic structures of samples within classes by some unsupervised clustering methods and directly embedding the structural information into the SVM objective function [72]. SSVM is theoretically and empirically a better generalization than SVM algorithm.

### 5.4. D Morphology

Three-dimensional morphology using 3D reconstruction and image processing techniques is applied for quantitative morphometric analysis of cellular and subcellular structures, which is much more powerful than its 2D counterpart, but still largely based on the processing of separate 2D slices.

The approach to 3D morphological analysis consists of digital micrographs acquisition, reconstruction, and 3D-based feature extraction. The acquired images are serialy taken by CT instrument at uniform angular intervals during a full 360° rotation [1], from the electron imaging film taken by photo products [73], or by electron microscopy [40]. Computer programs such as MATLAB or Visual Studio software can be used for automated 3D image reconstruction.

Based on the reconstructed models, features such as three-dimensional shape of the cells can be extracted, which are correlated with the assembly state of myofibrils in different stages [74] and ultrastructure such as the arrangement of compact chromatin of GO lymphocytes can be studied [23].

## 6. Existing Problems and Future Trends

Although morphological cell analysis has been developed in many applications as mature approaches for estimation and diagnosis, some problems still exist in its applications in biomedical engineering. Researchers are exerting efforts not only in simple localization and segmentation, but also in improving the methods mainly in the following aspects.

### 6.1. Real-Time Application and Computational Complexity

Morphological cell analysis has been applied in almost all hospitals, which are key means in automatic microscopic analysis. However, because of its high computational complexity, it has strict limits on the number and stability of feature points. The traditional method selects a few features, which limits the application scope of morphological analysis. The computational complexity greatly affects real-time application systems [50, 75].

### 6.2. Reliability

Reliability is a great concern in practical applications [55, 76]. Morphological analysis relies on tuning of many parameters. Related techniques rely on existing noise statistics, initial positions, and sufficiently good approximation of measurement functions. Deviations from such assumptions usually lead to degraded estimations during automatic analysis. Stochastic stability is established in terms of the conditions of the initial errors, bound on observation noise covariance, observation nonlinearity, and modeling error. Features have to be effectively and efficiently treated by their removal from or addition to the system. New methods should be explored to discard outliers and improve the matching rate. These will help stabilize algorithms and allow more accurate localizations or parametric estimations.

### 6.3. With a Priori Knowledge

Constraints introduced in morphological cell parameters may help in some occasions. For example, morphological cell analysis is commonly used to estimate the cell shapes and activities, which incorporate *a priori* information in a consistent manner. However, the known model or information are often either ignored or heuristically dealt with [6].

### 6.4. Accuracy

Accuracy is always the most important factor in biomedical engineering. The accuracy of the calculated cells strongly depends on the computational potential and the statistical possibilities. For example, automated method provides accurate segmentation of the cellular membranes in the stained tracts and reconstructs the approximate location of the unstained tracts using nuclear membranes as a spatial reference. Accurate cell-by-cell membrane segmentation allows per-cell morphological analysis and quantification of the target membrane [16, 51, 77].

### 6.5. Artificial Intelligence

The integration of the morphological cell analysis with some artificial intelligence methods may yield a better performance. Fuzzy logic, neural network, genetic algorithm, and so forth can be combined to wholly resolve the complex task.

## 7. Conclusion

This paper summarizes recent advances in morphological cell analysis for biomedical engineering applications. Typical contributions are addressed for initialization, localization, segmentation, estimation, modeling, shape analysis, cell parameters, and so forth. Representative works are listed for readers to have a general overview of state-of-the art. A number of methods for solving morphological problems are investigated. Many methods developed for morphological cell analysis, extended morphological cell segmentation, are introduced. In the 20-year history of morphological cell analysis, they gained entry into the field of biomedical engineering as a critical role. The largest volume of published reports in this literature belongs to the last ten years.

## Figures and Tables

**Table 1:** Representative contributions.

Processing	Method	Representative
Segmentation	Active contour model (ACM)	[5]—2011
	Reconstruct the approximate location of cellular membranes	[51]—2011
	A marker-controlled watershed transform and a snake model	[16]—2010
	Segmentation combing features	[51]—2011

Classification	*K*-means and support vector machines (SVM)	[6]—2011
	Bayesian classifier	[18]—2009

**Table 2:** Texture features.

Energy:	ASM = ∑_*i*=1_^*k*^(*g*_*i*_ − *g*)^−2^ *p*(*g*_*i*_)
Uniformity:	*U* = ∑_*i*=1_^*k*^ *p* ^2^(*g*_*i*_)

Entropy:	ENT = −∑_*i*=1_^*k*^ *p*(*g*_*i*_)log_2_ *p*(*g*_*i*_)

Smoothness:	IDM = 1 − 1/(1 + *s* ^2^), where $s=\sqrt{\sum_{i=1}^{k}{\left(g_{i}-g\right)}^{-2}p(g_{i})}$

Given that *g*_*i*_ is the gray value, *k* is the number of gray levels.
